# Preliminary Study on Clusterin Protein (sCLU) Expression in PC-12 Cells Overexpressing Wild-Type and Mutated (Swedish) *AβPP* genes Affected by Non-Steroid Isoprenoids and Water-Soluble Cholesterol

**DOI:** 10.3390/ijms20061481

**Published:** 2019-03-24

**Authors:** Beata Pająk, Elżbieta Kania, Anita Gołaszewska, Arkadiusz Orzechowski

**Affiliations:** 1 Independent Laboratory of Genetics and Molecular Biology, Kaczkowski Military Institute of Hygiene and Epidemiology, Kozielska 4, 01-163 Warsaw, Poland; bepaj@wp.pl; 2 Tumor Cell Death Laboratory, Cancer Research UK, Beatson Institute, Garscube Estate, Switchback Road, Glasgow G61 1BD, UK; elakania@gmail.com; 3 Department of Neuroendocrinology, Centre of Postgraduate Medical Education, Marymoncka 99/103, 01-813 Warsaw, Poland; an_ita@wp.pl; 4 Department of Physiological Sciences, Faculty of Veterinary Medicine, Warsaw University of Life Sciences – SGGW, Nowoursynowska 159, 02-776 Warsaw, Poland

**Keywords:** Alzheimer’s disease, statins, clusterin, cholesterol, mevalonate pathway, isoprenoids, rat neuronal pheochromocytoma PC-12 cells

## Abstract

In this study we attempted to verify the hypothesis that the mevalonate pathway affects amyloid beta precursor protein (AβPP) processing and regulates clusterin protein levels. *AβPP* expression was monitored by green fluorescence (FL) and Western blot (WB). WB showed soluble amyloid protein precursor alpha (sAβPPα) presence in *AβPP*-wt cells and Aβ expression in *AβPP*-sw cells. Nerve growth factor (NGF)-differentiated rat neuronal pheochromocytoma PC-12 cells were untreated/treated with statins alone or together with non-sterol isoprenoids. Co-treatment with mevalonate, dolichol, ubiquinol, farnesol, geranylgeraniol, or water-soluble cholesterol demonstrated statin-dependent neurotoxicity resulted from the attenuated activity of mevalonate pathway rather than lower cholesterol level. Atorvastatin (50 μM) or simvastatin (50 μM) as well as cholesterol chelator methyl-β-cyclodextrin (0.2 mM) diminished cell viability (*p* < 0.05) and clusterin levels. Interestingly, co-treatment with mevalonate, dolichol, ubiquinol, farnesol, geranylgeraniol, or water-soluble cholesterol stimulated (*p* < 0.05) clusterin expression. Effects of non-sterol isoprenoids, but not water soluble cholesterol (Chol-PEG), were the most significant in mock-transfected cells. Geranylgeraniol (GGOH) overcame atorvastatin (ATR)-dependent cytotoxicity. This effect does not seem to be dependent on clusterin, as its level became lower after GGOH. The novelty of these findings is that they show that the mevalonate (MEV) pathway rather than cholesterol itself plays an important role in clusterin expression levels. In mock-transfected, rather than in AβPP-overexpressing cells, GGOH/farnesol (FOH) exerted a protective effect. Thus, protein prenylation with GGOH/FOH might play substantial role in neuronal cell survival.

## 1. Introduction

Cholesterol synthesis and non-sterol isoprenoids play a vital role during the development of the central nervous system (CNS) [1,2]. Cholesterogenesis in the brain is fully autonomous because the blood brain barrier (BBB) obstructs plasma lipoproteins from entry into the cerebrospinal fluid (CSF). Interestingly, unesterified cholesterol is found mainly in myelin sheets and the plasma membrane of both neurons and glia, although the latter are the chief neuron suppliers. In the adult brain there is dynamic exchange of cholesterol complexed to apolipoprotein E between astrocytes (donors) and neurons (acceptors) [3]. The bulk of the evidence, however, suggests that isoprenoids rather than cholesterol itself play a crucial role in the adult brain [4,5]. Isoprenoids are synthesized in the mevalonate (MEV) pathway alternatively/together with cholesterol whereby 3-hydroxy- 3-methylglutaryl coenzyme A reductase (HMGCR, E.C 1.1.1.34) is a step limiting enzyme, ubiquitously expressed in animal cells, and hampered by a group of hypercholesterolemia lowering drugs named statins. Statins have access to the brain either through their lipophilicity or active transport [6]. Isoprenoid synthesis starts with five carbon chain (5-C) isoprene formed from decarboxylation of mevalonate pyrophosphate (MEVPP) to form isopentenyl pyrophosphate (IPP)/dimethylallyl diphosphate (DMPP). This step is followed by several condensations of IPP/DMPP or other isoprenoids resulted in geranyl pyrophosphate (GPP, 10-C), farnesyl pyrophosphate (FPP, 15-C) or geranylgeranyl pyrophosphate (GGPP, C-20). FPP is at the branching point to cholesterol synthesis, where together with another FPP molecule it rises to squalene (C-30). Compared to other tissues, FPP and GPP levels are highest in the brain [7]. Taken together, statins inhibit the MEV pathway, which plays a pivotal role in de novo cholesterol and isoprenoid synthesis. It is a matter of debate which end-product of the MEV pathway and in what circumstances are vital for brain functions in health and disease. With regard to Alzheimer’s disease (AD) overexpressed amyloid beta precursor protein (AβPP), a substrate for amyloid beta (Aβ) synthesis, was reported to suppress HMGCR [8].

Clusterin, also known as Apoliprotein J, the glycoprotein secreted (sCLU) mainly by neurons and glia, is widely recognized as extracellular chaperone with multifunctional properties including cell aggregation and inhibition of membrane attack complex in complement cascade [9]. Clusterin expression levels are higher in the brain in comparison to many other tissues [10,11]. sCLU occupies an original role in AD as it is involved in oxidative stress, inflammatory response and clearance of Aβ from brain [12,13]. Moreover, sCLU is believed to suppress immune reaction, as it is induced through toll like receptor (TLR3) signaling to inhibit nuclear factor kappa-light-chain-enhancer of activated B cells (NF-κB), the transcription factor controlling key functions of immune cells [14]. There are other proofs that sCLU is involved in immune tolerance, as it was demonstrated to limit myocarditis [15] and trouble-free removal of dead cells and cell debris [16].

Clusterin (*CLU*) gene expression is observed in damaged tissue and it is unevenly distributed with some grey matter areas of higher representation in the rat brain [17]. *CLU* mRNA is induced by an at present unknown signaling pathway through plasma membrane phospholipid phosphatidylserine (PS), playing a major role as a marker of apoptotic and necrotic cells [16]. The role of CLU in neuroprotection is apparently equivocal [18]. Anyway, experimental data show sCLU seems to fulfill the role of extracellular chaperone by promoting the disposal of dead cells and cell remnants [19]. Whether sCLU protein assists in the “nonprofessional” phagocytosis mediated by epithelial, endothelial, fibroblast and smooth muscle cells is a matter of debate, even though sCLU has the ability to bind a broad spectrum of proteins playing the role of the docking platform for cellular uptake [20].

sCLU may also play a role in transport/uptake vehicle of amyloid beta (Aβ) in AD [12,13]. Several cohort studies and meta-analyses suggest that *CLU* gene rs11136000 variant is significantly associated with Alzheimer’s disease [21,22,23]. Numerous papers report higher clusterin expression in the brains affected by AD [24,25,26]. It colocalizes with Aβ, the product of subsequent AβPP processing by β- (BACE1) and γ-secretase, suggesting the central role played by this protein in senile plaque formation [9,26,27]. sCLU was shown to inhibit the aggregation [28] while promoting evacuation of Aβ through the blood brain barrier (BBB) [25,29]. The latter event most likely occurs through CLU Aβ_42_-induced endocytosis and accumulation in astrocytes [30,31]. Furthermore, *CLU rs11136000* single nucleotide polymorphism (SNP) modified the cerebrospinal fluid (CSF) levels of the microtubule-associated protein Tau in AD patients [32]. Furthermore, intracellular clusterin (iCLU) was upregulated in the brain of Tau overexpressing Tg4510 mice. There are some reports pointing to oxidative stress induced by sCLU-Aβ complexes [27,28], while others emphasize binding of Aβ as the indirect cytoprotective mechanism of Aβ clearance and transport [33,34]. Importantly, clusterin protein concentration paralleled mRNA expression, and this protein was suggested to be a good marker of cell senescence [35,36]. Physiological mechanisms of Aβ clearance are controlled on one hand by extracellular degradation through neprilysin and insulin-degrading enzymes, on the other hand by astrocytes and microglia via endocytotic/phagocytotic pathways [37,38]. Aβ clearance from brain to blood by transcytosis across the BBB is possible only if the peptide is bound to apolipoprotein E (apoE), α2-macroglobulin (α2M) or sCLU. The latter (1:1 sCLU-Aβ complex binds to lipoprotein low density-receptor-related protein 2 (LRP-2/megalin receptor) expressed in endothelium, ependyma and choroid plexus, whereas the apoE-Aβ and α2M-Aβ complexes need LRP-1 [12,39]. The opposite, Aβ transport from blood to brain via BBB, is mediated by receptors for advanced glycation end products (RAGE), thereby highlighting the importance of respective receptor balance in Aβ brain deposition. As demonstrated by others, sCLU might play important role in the endocytosis/autophagy as astrocytes loaded with fibrillar Aβ had upregulated sCLU expression levels [30]. Cells are induced to form cytoplasmic vacuoles, presumably due to uptake of sCLU-Aβ complexes, pointing to sCLU as critical extracellular component regulating Aβ clearance from the brain. Previously, we showed that PC-12 neuronal cells with *AβPP*-sw (Swedish mutation) but not that of *AβPP*-wt gene or empty vector had enhanced representation of autophagy-like vacuoles [40].

In the present study we investigated whether increased Aβ production in *AβPP*-sw expressing PC-12 neuronal cells is paralleled by enhanced expression of sCLU. Additionally, we wished to know how Aβ processing in *AβPP*-sw expressing cells affects cell viability. To examine this in the context of how important the MEV pathway is, we tested several intermediates including non-sterol isoprenoids and water-soluble cholesterol, to see whether any of the substance can prevent neuronal cells from atorvastatin- or simvastatin-induced fall in cell viability. We also tested the effect of selective inhibitors of MEV pathway enzymes on viability measurement of neuronal cells.

## 2. Results

### 2.1. Effects of Selected Statins or MβCD on Cell Viability of Neuronal PC-12 Cells Overexpressing Wild-Type or Mutated (Swedish Mutation) AβPP Gene

**Figure 1:**
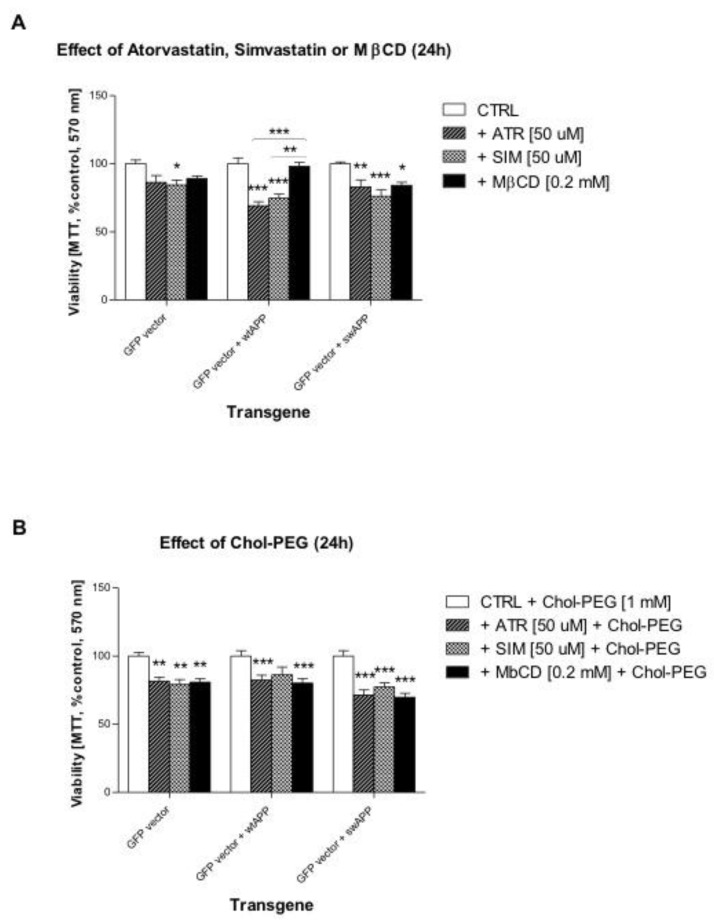
Effect of water-soluble cholesterol (Chol-PEG, 1 mM) on cell viability affected by mevalonate (MEV) pathway modulators (atorvastatin – ATR, simvastatin – SIM, 50 μM each) or cholesterol chelator methyl-β-cyclodextrin (MβCD, 0.2 mM). One day (24 h) treatment with (**A**) ATR or SIM (50 μM) or MβCD (0.2 mM) alone or (**B**) together with Chol-PEG. Bar charts show percentage (% control) cell viability measured by [3-(4,5-dimethylthiazol-2-yl)-2-5-diphenyltetrazolium bromide] (MTT) assay. Two-way ANOVA test followed by Bonferroni’s multiple comparisons was employed to analyze the data. (**A**) ATR, SIM or MβCD dose-dependently diminished fraction of viable cells. Except MβCD, neither *AβPP*-wt, nor Swedish mutation *AβPP*-sw gene affected ATR- or SIM-dependent effects on viable cells in comparison to empty-vector transfected cells. The results amounted to: *p* < 0.0302 for interaction, *p* < 0.1409 for genes, *p* < 0.0001 for ATR; *p* < 0.0629 for interaction, *p* < 0.338 for genes, *p* < 0.0001 for SIM; *p* < 0.0343 for interaction, *p* < 0.0024 for genes, *p* < 0.0667 for MβCD. (**B**) Adding Chol-PEG could not rescue ATR, or SIM, or MβCD diminished viable cells. Both *AβPP*-wt and Swedish mutation *AβPP*-sw genes affected Chol-PEG-dependent effects on viable cells in comparison to empty-vector transfected cells. The results amounted to: *p* = 0.5034 for interaction, *p* < 0.0117 for genes, *p* < 0.0001 for Chol-PEG. Error bars = S.E.M., and * *p* < 0.05, ** *p* < 0.01, *** *p* < 0.001 for comparison with non-treated control cells. The results are indicative of three independent experiments performed in eight replicates.

Atorvastatin (50 μM, ATR), simvastatin (50 μM, SIM) or methyl-β-cyclodextrin (0.2 mM, MβCD) were used in one-day treatment (24 h) in PC-12 neuronal cells with *AβPP*-wt, *AβPP*-sw or mock-transfected cells. This experiment demonstrated higher susceptibility (lower cell viability) of *AβPP*-wt transfected cells to HMGCR inhibitors, but not to MβCD when compared to non-treated control cells (*p* < 0.001, Figure 1A). Additionally, *AβPP*-sw gene rendered PC-12 neuronal cells highly vulnerable to any statin or MβCD (*p* < 0.05–0.001, Figure 1A). To reverse the effects of ATR, SIM or MβCD, which caused cholesterol depletion, water soluble cholesterol (1 mM, Chol-PEG) was co-administered. The protective effect of Chol-PEG was hardly observed, it does evenly strengthen MβCD-induced loss in cell viability with regard to non-treated control cells (*p* < 0.001, Figure 1B).

### 2.2. Effect of Some Non-Sterol Isoprenoids on Cell Viability of Neuronal PC-12 Cells Overexpressing Wild-Type or Mutated (Swedish Mutation) AβPP Gene Treated with Selected Statins or MβCD

Statins inhibit HMGCR, the rate-limiting enzyme of the MEV pathway. As HMGCR inhibitors, statins limit MEV entry for several intermediary metabolic pathways, such as synthesis of non-sterol isoprenoids (geranylgeraniol, farnesol, dolichol, ubiquinol), as well as cholesterol. The effects of different non-sterol isoprenoids were tested herein (mevalonate – MEV 100 μM, geranylgeraniol – GGOH 10 μM, farnesol – FOH 10 μM, dolichol – DOH 0.1 μg/mL, ubiquinol – UBOH 10 μg/mL) vs. cholesterol as water soluble conjugate Chol-PEG (1 mM) in cell cultures challenged with statins or cholesterol chelator methyl-β-cyclodextrin (MβCD, Supplementary data 2). Metabolic inhibitors of selected enzymes in the MEV pathway were also monitored via the [3-(4,5-dimethylthiazol-2-yl)-2-5-diphenyltetrazolium bromide] (MTT) assay (Supplementary data 3).

**Figure 2:**
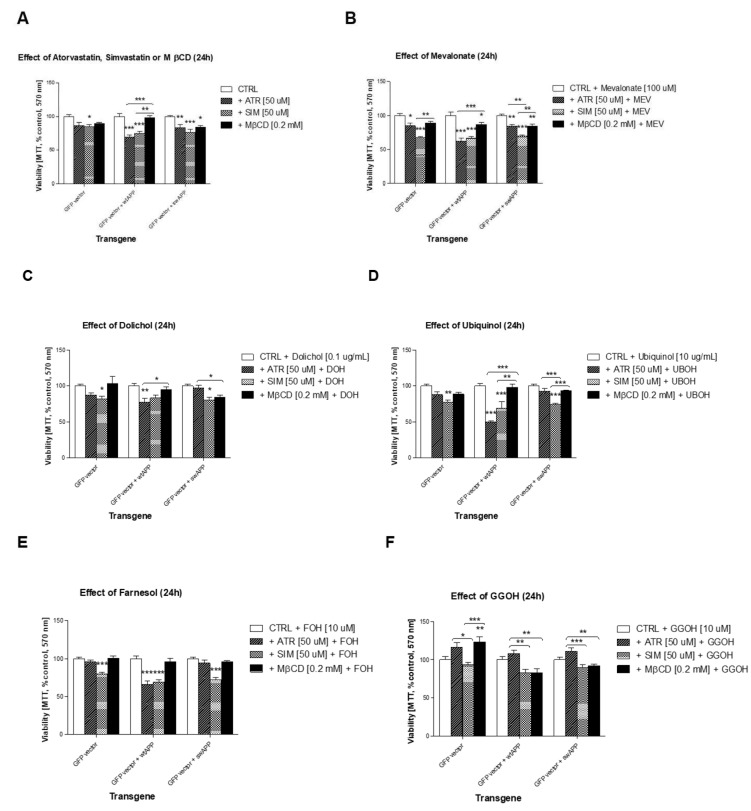
Effect of mevalonate (MEV, 100 μM), dolichol (DOH, 0.1 μg/mL), ubiquinol (UBOH, 10 μg/mL), farnesol (FOH, 10 μM) or geranylgeraniol (GGOH, 10 μM) on cell viability affected by MEV pathway modulators (atorvastatin – ATR, simvastatin – SIM, 50 μM each) or cholesterol chelator methyl-β-cyclodextrin (MβCD, 0.2 mM). One day (24 h) treatment with (**A**) ATR or SIM (50 μM) or MβCD (0.2 mM) alone or (**B**) together with MEV, (**C**) or with DOH, (**D**) or with UBOH. Bar charts show percentage (% control) cell viability measured by MTT assay. Two-way ANOVA test followed by Bonferroni’s multiple comparisons were employed to analyze the data. (**A**) ATR, SIM or MβCD dose-dependently diminished fraction of viable cells. Except MβCD, neither *AβPP*-wt, nor Swedish mutation *AβPP*-sw gene affected ATR- or SIM-dependent effects on viable cells in comparison to empty-vector transfected cells. The results amounted to: *p* < 0.0302 for interaction, *p* < 0.1409 for genes, *p* < 0.0001 for ATR; *p* < 0.0629 for interaction, *p* < 0.338 for genes, *p* < 0.0001 for SIM; *p* < 0.0343 for interaction, *p* < 0.0024 for genes, *p* < 0.0667 for MβCD. (**B**) Adding MEV could rescue ATR, or SIM, or MβCD diminished viable cells. Both *AβPP*-wt and Swedish mutation *AβPP*-sw genes affected MEV-dependent effects on viable cells in comparison to empty-vector transfected cells. The results amounted to: *p* < 0.016 for interaction, *p* < 0.0112 for genes, *p* < 0.0001 for MEV. (**C**) Adding DOH rescued ATR, or SIM, or MβCD diminished viable cells. Neither *AβPP*-wt, nor Swedish mutation *AβPP*-sw gene affected DOH-dependent effects on viable cells in comparison to empty-vector transfected cells. The results amounted to: *p* < 0.0153 for interaction, *p* < 0.4076 for genes, *p* < 0.0001 for DOH. (**D**) Adding UBOH rescued ATR, or SIM, or MβCD diminished viable cells. Both *AβPP*-wt and Swedish mutation *AβPP*-sw genes affected UBOH-dependent effects on viable cells in comparison to empty-vector transfected cells. The results amounted to: *p* < 0.0001 for interaction, *p* = 0.0004 for genes, *p* < 0.0001 for UBOH. (**E**) Adding FOH rescued ATR, or SIM, or MβCD diminished viable cells. Both *AβPP*-wt and Swedish mutation *AβPP*-sw genes affected UBOH-dependent effects on viable cells in comparison to empty-vector transfected cells. The results amounted to: *p* < 0.0001 for interaction, *p* < 0.0001 for genes, *p* < 0.0001 for FOH. (**F**) Adding GGOH rescued ATR, or SIM, or MβCD diminished viable cells. Both *AβPP*-wt and Swedish mutation *AβPP*-sw genes affected GGOH-dependent effects on viable cells in comparison to empty-vector transfected cells. The results amounted to: *p* = 0.0004 for interaction, *p* < 0.0001 for genes, *p* < 0.0001 for GGOH; Error bars = S.E.M., and * *p* < 0.05, ** *p* < 0.01, *** *p* < 0.001 for comparison with non-treated control cells. The results are indicative of three independent experiments performed in eight replicates.

Initially, the cytoprotective effect of MEV pathway intermediates was evaluated. Full reversal of ATR-, and to some extent SIM- or MβCD-induced cytotoxicity was observed in PC-12 neuronal cells with *AβPP*-wt, *AβPP*-sw or empty vector after administration of 10 μM GGOH (Figure 2F). The cytoprotective effect of GGOH against ATR-induced cytotoxicity was significant, or highly significant in comparison to SIM- or MβCD-treated cells (Figure 2F). Astonishingly, no other non-sterol isoprenoid, except FOH, mimicked GGOH in reversal any statin- or MβCD-dependent loss in cell viability although some co-treatments (MEV, DOH, UBOH) led to statistically significant differences in average viability between *AβPP*-wt, (W), and *AβPP*-sw, (S) transfected cells (Figure 2B–2E). FOH, similarly to GGOH, reversed ATR- and MβCD-toxicity of *AβPP*-sw cells to control levels (Figure 2E, *p* > 0.05). Figure 1A and 2A are the same for clearer demonstration of differences in cell viability between statins or MβCD given alone or co-treated with soluble cholesterol vs. MEV pathway intermediates.

### 2.3. Effects of Selected MEV Pathway Enzyme Inhibitors on Cell Viability of Neuronal PC-12 Cells Overexpressing Wild-Type and Mutated (Swedish Mutation) AβPP Gene

To gain insight into the cellular pathways translating into the reduced cell viability depicted in Figure 1, additional analysis with selected MEV pathway enzyme inhibitors was carried out. As illustrated in Supplementary data 3, zaragozic acid (squalene synthase inhibitor), L-744,832 (farnesyl transferase inhibitor), or GGTI-286 (geranylgeranyl transferase I inhibitor) dose-dependently reduced cell viability similarly to statins or MβCD. These observations confirm the importance of the inhibited steps in MEV pathway for neuronal cell survival regardless of transfected gene. Next, we sought which intermediate of MEV pathway is essential for viable cell population impeded by statins or MβCD.

### 2.4. Effects of Non-Sterol Isoprenoids (MEV, DOH, UBOH, FOH, GGOH) vs. CHOL-PEG on β-Amyloid (Aβ1-40) Secretion by Neuronal PC-12 Cells Affected by MEV Pathway Modulators Statins or Cholesterol Chelator Methyl-β-Cyclodextrin (MβCD) in PC-12 Cells Overexpressing AβPP-wt or AβPP-sw Gene

**Figure 3:**
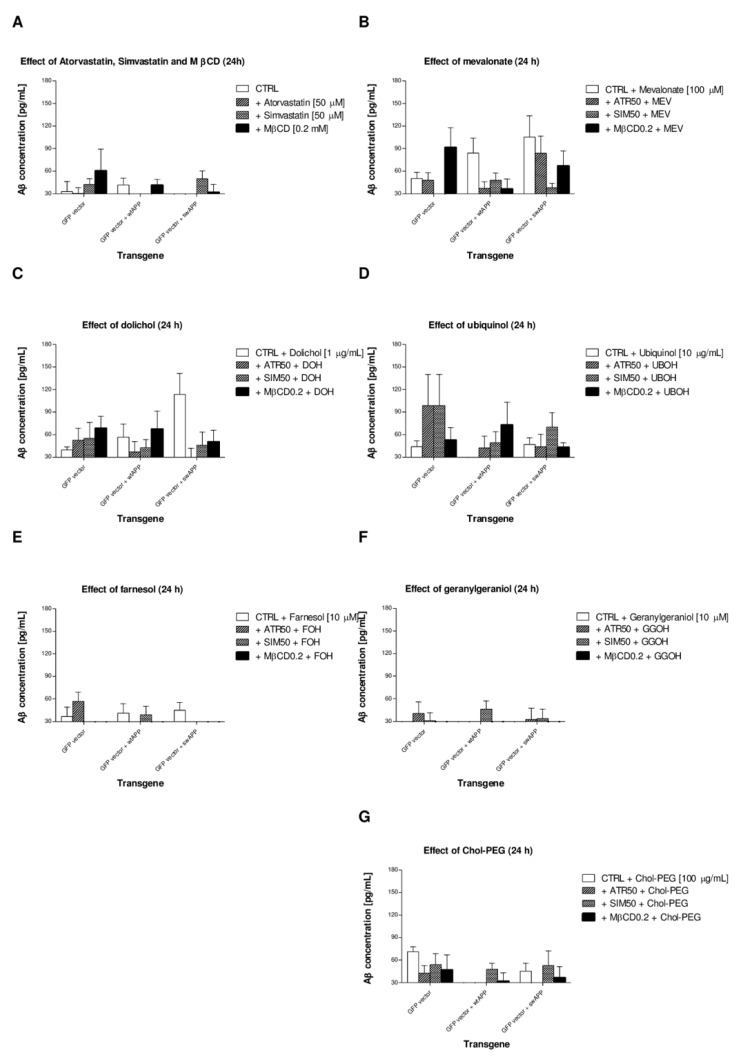
Effect of mevalonate (MEV, 100 μM), dolichol (DOH, 0.1 μg/mL), ubiquinol (UBOH, 10 μg/mL), farnesol (FOH, 10 μM), geranylgeraniol (GGOH, 10 μM) or water soluble cholesterol (Chol-PEG, 1 mM) on amyloid-β (Aβ_40_) secretion by neurnal PC-12 cells affected by MEV pathway modulators (atorvastatin – ATR, simvastatin – SIM, 50 μM each) or cholesterol chelator methyl-β-cyclodextrin (MβCD, 0.2 mM). Concentration of Aβ1–40 was determined by homogenous time resolved fluorescence (HTRF) assay (CisBio) according to the manufacturer’s instructions as described in the Materials & Methods section. Subsequent figures (**A**–**G**) represent Aβ concentration in culture media collected and determined after experiment was completed and corrected for cell viability.

The average concentrations of amyloid (Aβ1-40) in supernatants collected from treatments shown on Figure 2 points to fairly low amyloidogenic AβPP processing in neuronal PC-12 cells upon statins (ATR, SIM, 50 μM) or MβCD (0.2 mM) treatments (Figure 3A). The Aβ production increased after additional co-treatment with MEV, DOH or UBOH (Figure 3B–D), but it returned to control levels after co-treatment with FOH, GGOH or Chol-PEG (Figure 3E–G). These data point to the central role played by protein prenylation and cholesterol itself in controlling the activity of amyloidogenic AβPP processing.

Cellular media were harvested and assayed for the presence of Aβ_40_ normalized for cell viability, as determined with the MTT assay. Experiments were performed at least three times, and each point in each experiment has eight repetitions.

### 2.5. Effects of Mevalonate (MEV, FOH, GGOH) or Water-Soluble Cholesterol (Chol-PEG) on Selected Protein Expression Levels in Cells Affected by MEV Pathway Modulators (Atorvastatin – ATR, Simvastatin – SIM)

**Figure 4:**
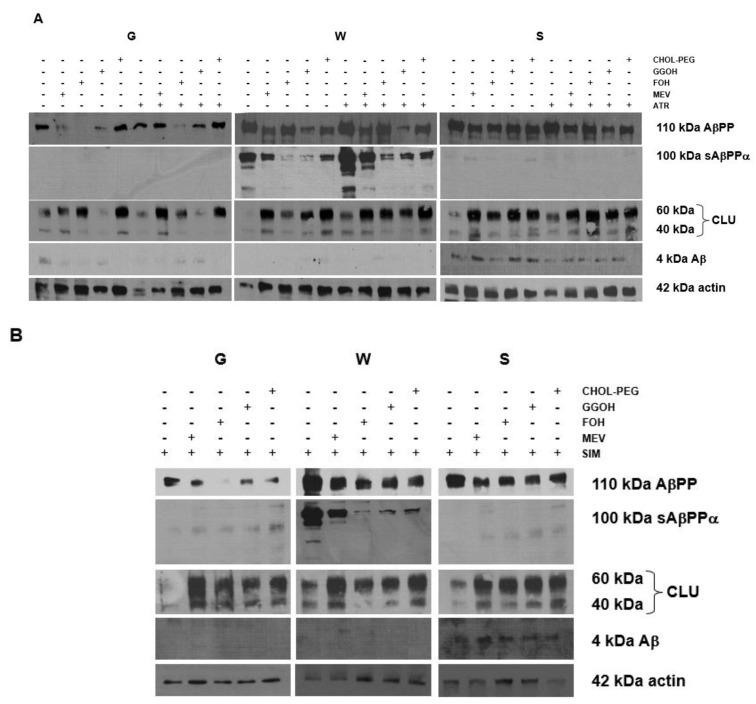
Effect of mevalonate (MEV, 100 μM), farnesol (FOH, 10 μM), geranylgeraniol (GGOH, 10 μM) or water soluble cholesterol (Chol-PEG, 1 mM) on selected protein expressions in cells affected by MEV pathway modulators (**A** for atorvastatin – ATR, **B** for simvastatin – SIM). Western blot analysis of proteins: amyloid precursor protein (AβPP, 110 kDa), soluble amyloid protein precursor alpha (sAβPPα, 100 kDa), clusterin (CLU, 60 and 40 kDa), amyloid-β 1-16 (Aβ, 4 kDa), actin (42 kDa) as house-keeping protein in empty vector (G), *AβPP* wild type gene (*AβPP*-wt, W), and *AβPP* Swedish mutations gene (*AβPP*-sw, S) transfected cells. The results are indicative of three independent experiments with similar results.

As neuronal cells are most likely targeted by statins rather than MβCD, we sought the impact of ATR or SIM (50 μM each), as well as co-treatment with selected non-sterol isoprenoids, on AβPP amyloidogenic processing. First, amyloidogenic AβPP processing was observed ultimately in *AβPP*-sw-, whereas non-amyloidogenic AβPP cleavage was found exclusively in neuronal cells bearing *AβPP*-wt (Figure 4A,B). Co-treatment with non-sterol isoprenoids GGOH (10 μM), FOH (10 μM), MEV (100 μM) and Chol-PEG (1 mM) in non-toxic concentrations strongly stimulated (*p* < 0.05) clusterin protein levels in PC-12 neuronal cells. The effects of co-treatment with non-sterol isoprenoids, but not Chol-PEG, were more profound in empty-vector than in *AβPP*-wt and *AβPP*-sw transfected cells, showing the high importance of protein prenylation in neuronal cells.

## 3. Discussion

Previously, we reported that Swedish mutation *AβPP*-sw under control of *GFP* gene promoter reduced viability in NGF-induced neuronal PC-12 cells compared to empty-vector transfected cells with symptoms of aberrant autophagy and increased clusterin protein expression [40]. We showed the incomplete autophagy-like process induced by human *AβPP*-sw gene inserted in neuronal PC-12 cells. The evidence of excess autophagosomes and multivesicular bodies found in *AβPP*-sw- but not in *AβPP*-wt- or empty vector-bearing neuronal cells was accompanied by elevated sCLU expression levels. In this work we tried to figure out how the MEV pathway alters *AβPP*-sw gene dependent sCLU protein expression levels. We report here that, compared to non-treated cells, the viability of *AβPP*-sw- and *AβPP*-wt-transfected neuronal PC-12 cells exemplified on respective photographs (Supplementary data 1) was similarly reduced by statins and MβCD (Supplementary data 2, Figure 1). Chol-PEG administration could hardly restore cell viability diminished by ATR, SIM or MβCD (Figure 1, *p* > 0.05) so we assumed that diminished neuronal cell survival by statins or MβCD could be independent of a reduction in cholesterol level. We also tested the effects of metabolic inhibitors of MEV pathway on cell viability. As shown in respective plots (Supplementary data 3), the inhibitors of squalene synthase (SQS) - zaragozic acid [10–100 μM], farnesyl transferase – L-744,832 [10–100 μM], and geranylgeranyl transferase I – GGTI-286 [1–10 μM] decreased significantly the viability of neuronal PC-12 cells expressing the Alzheimer pertinent protein AβPP695NL (*AβPP*-sw-transfected cells).

These data point to the important role played by the MEV pathway in neuroprotection other than cholesterol itself. Recently, some authors have highlighted the role of non-sterol isoprenoids in neurons, suggesting that there is a shift of the MEV pathway towards the non-sterol isoprenoids which are essential for synaptic activity [41]. In a process known as prenylation, isoprenoids are covalently bound to small guanosine triphosphate binding proteins (sGTPases) of primary importance for neurons [42]. Among them, Rab3 and RhoA are involved in neurotransmitter release, and synaptic plasticity, respectively [41]. Statins, including simvastatin, cause marked drop in RhoA prenylation which is associated with Akt kinase phosphorylation (activation) followed by cAMP responsive element binding protein 1 (CREB 1) activation. In consequence, this transcription factor upregulates cognition and memory by genomic regulation of *c-Fos* and *Egr-1* gene transcription [43,44]. Thus, depending on type and dose of statin, it may play either a beneficial or harmful role in brain function.

As a matter of fact, we decided to test if MEV pathway intermediates could prevent ATR-, SIM- or MβCD-retarded cell viability. Cell viability was used as an index to evaluate cytoprotective/cytotoxic attributes of particular substance. Prosurvival activity was demonstrated for GGOH when this non-sterol isoprenoid was added to ATR-, SIM- or MβCD-treated cells irrespective of inserted gene (Figure 2). Noticeably, GGOH prevented ATR cytotoxicity better than in SIM or MβCD treatments (Figure 2, *p* < 0.01–0.001), showing that protein prenylation with geranylgeraniol might be more impaired by ATR than SIM. Actually, several studies highlight the physiological significance of geranylgeranylation catalyzed by two types of enzymes (GGTase-I liable for monogeranylgeranylation, and GGTase-II that catalyzes digeranylgeranylation of sGTPases) [45,46]. The aforementioned effect of GGOH in PC-12 neuronal cells looks a lot like that described by us recently in C2C12 mouse myoblasts [47]. Neuronal survival is diminished by statins; moreover, statin effect is independent of reduced cholesterol level, since neuronal viability is restored by FPP/GGPP supplementation, and not by adding cholesterol [48]. Importantly, the long-term potentiation (LTP) known as long-lasting strengthening of synapses between nerve cells could be blocked with statins [49]. As a result, long-term memories are fully dependent on the MEV pathway and geranylgeraniol, but cholesterol is not able to reverse the LTP blockade [50]. For more details about links between the MEV pathway and AD see the papers [4,5,6,7]. In a similar way to our cell viability study, GGOH-dependent prenylation was demonstrated to increase neuronal dendritic outgrowth and dendritic protrusion density, as well as increased synaptic proteins in crude synaptosomal fractions [43]. By all means, the MEV pathway with GGOH preserve neuronal functions because prenylation of some sGTPases mediated by GGTase-I seems to maintain cognitive functions [51]. Since incubation with FOH alike GGOH prevented ATR-induced toxicity (Figure 2E) we admit FOH could contribute to GGOH production. It is plausible to expect FOH in the non-sterol pathway as the GGOH synthesis in neurons is favored over squalene [52]. Additionally, examination of gene expression patterns points to lower activity of the pre-squalene pathway in neurons [53], which favors the non-sterol MEV pathway, giving rise to FPP, GGPP, ubiquinone, dolichol or heme A. Altogether, these data and recalled reports advocate boosted non-sterol branch of the MEV pathway in neurons, probably owing to the prenylation of sGTPases [54]. In muscle cells we found that sGTPase RAP1 is a good candidate to control cell viability during myogenesis from C2C12 mouse myoblasts [47]. It should be pointed out that regardless of a possible shutdown, the branch of squalene synthesis remains vital to neurons, as plasma membrane nanodomains (lipid rafts, LR) with its cholesterol and sphingolipids control cell signaling and protein processing [55].

Currently, several novel pathways are indicated in AD pathogenesis, including sGTPases. These proteins have been implicated as critical components in AD pathogenesis, although their cognate targets in AD remain obscure. Binding GTP activates sGTPases, while inactivation is achieved by GDP. Regulatory proteins regulate GDP/GTP exchange in sGTPases. The guanine nucleotide exchange factors (GEFs) support GTP bound state (activation) whereas GTPase activating proteins (GAPs) preserve GDP bound state (inactivation). AβPP and Aβ expression levels have been shown to increase in response to active sGTPases [56,57]. As sGTPases are not transmembrane proteins they require addition of FOH or GGOH at their C termini for normal GTPase function [54]. In contrast to MβCD, which depletes just plasma cholesterol, statins in turn lower the concentrations of FPP and GGPP, despite their cholesterol lowering effect. As a result, treatment with statins additionally inhibits the membrane anchorage of sGTPases and AβPP processing to AβPP C-terminal fragments and Aβ in neuroblastoma cells [57]. Previously [36], and here, we have demonstrated that AβPP processing is determined by the presence of an additional *AβPP* gene. AβPP C-terminal fragment (sAβPPα) stems from AβPP in the *AβPP*-wt-bearing cells, but could be found neither in empty-vector or cells having the *AβPP*-sw gene (Figure 4A,B). Contradictory to empty-vector and *AβPP*-wt-bearing cells, the Aβ peptide was expressed solely in *AβPP*-sw-bearing cells (Figure 4A,B). Overall it points to distinct mechanisms of AβPP processing in empty-vector, *AβPP*-wt-, and *AβPP*-sw-bearing cells. In addition, the expression of AβPP C-terminal fragment sAβPPα was markedly elevated in ATR- or SIM-treated *AβPP*-wt-bearing cells, while its expression was reduced by co-treatment with isoprenoids or Chol-PEG. Effects of isoprenoids given separately were similar with respect to Aβ expression levels compared to co-treatment with ATR or SIM in *AβPP*-sw-bearing cells (Figure 4A,B). ATR but not SIM administration inhibited Aβ expression (Figure 4A,B). Chol-PEG was incapable of reversing the ATR effect, which substantiates the idea that isoprenoids somehow control AβPP processing to Aβ. Isoprenylation of some sGTPases seems to be involved in AD, as Rab-6 GTPase levels are augmented in affected individuals and control presenilin 1, which in turn assists γ-secretase in Aβ formation [58,59]. Complementary to our findings, endogenous isoprenoids FPP and GGPP given to H4 neuroglioma cells expressing *AβPP*-sw gene increased Aβ production imitating AD, although different forms (pyrophosphates) and/or higher concentrations of isoprenoids were used by authors [60].

Our observations suggest that homeostasis of non-sterol isoprenoids is of higher importance than cholesterol itself in *AβPP*-sw-bearing cells. These data differ from reports of some authors [56,57,61], but are in agreement with others [3,60,62]. It is worth noting that amplifying the MEV pathway is cytoprotective via upstream farnesyl transferase (FTase) and/or geranylgeranyl transferases (GGTases), rather than squalene synthase (SQS) once GGTase I is fully operational, which was demonstrated in skeletal muscle cells [47]. What comes up to the sight is that enzyme activity of the MEV pathway should be met by substrate delivery, since inhibition of GGTase I resulted in GGOH cytotoxic side-effects [47]. Thus, the balance between MEV pathway activity and substrate fluxes has a fundamental connotation, as a brain with elevated GGPP exhibited lower mRNA and GGTase I activity [51]. This idea is substantiated by the elevated concentrations of FPP and GGPP determined in post-mortem AD brains compared to healthy brains [63]. Previously, similar findings were reported for dolichol and ubiquinone levels, which rose in elderly and AD brains [64,65,66]. If, as some authors claim, statins could be regarded as drugs for AD [57,63], it raises concern that inhibition of HMGCR affects the isoprenoids more than cholesterol. There is no clear evidence that statins exhibit any beneficial effect on amyloid plaque density at autopsy, although use of statins by healthy elderly individuals correlated with significant reduction of Tau protein in cerebrospinal fluid (CSF) [67] and brain neurofibrillary tangles (NFT) density compared to statin nonusers [68].

Accordingly, the aberrant protein prenylation has a greater effect on AD than cholesterol itself [62]. Most recent experiments bring to light cholesterol metabolism in pathology of sporadic AD (late-onset Alzheimer’s disease, LOAD), as well as genes related to sterol metabolism [69,70]. It is clear from these reports that phosphorylated Tau (p-Tau) protein level and NFT density in the frontal cortex resulted from increased FPP and GGPP synthesis, and that sterol regulatory element binding transcription factor 2 (*SREBF2*) gene expression levels in LOAD frontal cortices were inversely related to age of death.

In contrast to immunoblotting, we could not demonstrate that the rise in Aβ concentration in medium collected from treatments came with the highest Aβ expression levels determined by Western blotting in cell lysates (Figure 3). Moreover, the Aβ concentration was lowest upon FOH or GGOH treatment, also in PC-12 cells expressing the Alzheimer relevant protein AβPP695NL (*AβPP*-sw-bearing cells) (Figure 3E,F). The highest Aβ concentration was found in medium collected from MEV co-treatment (Figure 3B). This inconsistency could stem from Aβ intracellular preservation, which is common in different neuronal cell types examined [71,72]. Immunocytochemistry combined with quantitative image analysis would be helpful to corroborate this hypothesis, since clear-cut revelation of intracellular Aβ is inadequate. It is worth noting that intracellular accumulation of Aβ precedes its extracellular export [73,74,75,76] and causes synaptic pathology in AD associated with aberrant autophagy [40,76,77].

This study shows for the first time that CLU protein expression levels are inversely affected by statins which are inhibitors whereas non-sterol isoprenoids or water-soluble cholesterol are stimulators of this protein synthesis (Figure 4). Moreover, in empty-vector PC-12 neuronal cells (G) but not in those expressing sAβPPα (W) or Aβ (S) the administration of GGOH reduced CLU expression to control levels (Figure 4A). As CLU secretion is known to occur in inflammation, we suggest that GGOH could repress the inflammatory response in AβPP expressing cells. This effect of GGOH is markedly reduced in *AβPP*-wt- and *AβPP*-sw-bearing cells. Therefore, expression of sAβPPα (W) or Aβ (S) has a considerable impact on CLU protein expression levels.

To shed the light on the neuroprotective role of GGOH/FOH in the survival of neuronal cells, it is tempting to follow reports from mevalonate kinase gene (*MVK*) deficiency (MKD). This is a rare enzymatic defect that is biochemically reproduced by the use of statins, the competitive HMGCR inhibitors. In a number of studies, it was demonstrated that MKD is associated with lower neuronal cell viability caused by inflammation, defective autophagy, oxidative stress, and programmed cell death [78,79,80]. Interestingly, cell death induced by the MEV pathway inhibitor lovastatin in SH-SY5Y cells was hampered by GGOH co-treatment [80], which is in agreement with our results, as the same isoprenoid restored viability reduced by ATR or SIM in PC-12 cells. Consequently, either MKD or statin-dependent blocking of the MEV pathway lead to oxidative stress, inflammatory response and apoptosis [81,82,83]. One possible scenario for apoptosis in MKD or statin-dependent blocking of the MEV pathway is defective autophagy, as autophagy which rescues cells from different insults is highly dependent on prenylated sGTPases [84,85]. When autophagy is compromised via prenylation deficit of sGTPases, apoptosis takes over and cells undergo deletion. A body of evidence suggests that mitochondria are targeted by MKD or statins and that mitochondrial dysfunction is linked to apoptosis [79,80,86]. In addition, these spoiled mitochondria are not efficiently targeted for degradation in statin-treated cells showing autophagy (mitophagy) impairment followed by oxidative stress, apoptosis, DNA damage and cellular stress [78,87]. Currently, we are undertaking an ongoing study centered on mitochondria, as these organelle exhibit drops in mitochondrial membrane potential (MMP) to statin treatment which is reversed by GGOH co-treatment [88].

In summary, we have to stress that, to our knowledge, this is the first report showing sCLU expression stimulated by isoprenoids and water-soluble cholesterol; however, it was not the case for GGOH administration in empty-vector cells. Statins, inhibitors of FTase, GGTase I, and SQS, as well as cholesterol chelator reduced viability of neuronal PC-12 cells. As the MEV pathway is vital for life functions in eukaryotic cells we suggest that the defective mechanism of HMGCR induced by statins is overruled by GGOH, but not cholesterol administration. In the past, similar observations were reported by Campia et al. [89] from examination of THP-1 cells. Besides, if sCLU secretion is believed to be a good biomarker of inflammatory response, the lack of response to GGOH in empty-vector cells confirms the assumption that sAβPPα (W) or Aβ (S) are connected to inflammation in neuronal cells. Inflammatory response (not determined in our study) was previously reported to occur in neuronal cells with alteration of the MEV pathway, however, this reaction was repressed by isoprenoids but not cholesterol [90,91]. In cells with a debilitated MEV pathway, the mechanism that triggers inflammation is the enhanced expression of *NLPR3*-inflammasome, which in turn leads to damaged mitochondria [92,93]. In consequence, mitochondrion is to be the most likely target organelle by impaired cholesterol metabolism with the subsequent fall in cellular energy homeostasis, production of reactive oxygen/nitrogen species (ROS/NOS) in extreme cases leading to cell death by apoptosis. There is an urgent need to scrutinize the molecular mechanism of impaired mitochondrial function evoked by MEV pathway inhibitors as some results point to wrecked mitophagy in AD, which obviously plays a critical role in neuronal cell survival [94].

## 4. Materials and Methods

### 4.1. PC-12 Cell Culture

The rat PC-12 cell line was obtained from European Collection of Animal Cell Cultures (ECAAC). The cells were cultured, proliferated and differentiated as described elsewhere [40]. Briefly, cells were cultured as monolayer in growth medium (GM) Dulbecco’s Modified Eagle’s Medium with Glutamax (DMEM, Thermo Scientific, Paisley, Scotland) containing 10% (*v*/*v*) fetal bovine serum (FBS) + 5% (*v*/*v*) horse serum (HS, Gibco Life Technologies, New Zealand origin, Paisley, Scotland), antibiotics (penicillin:streptomycin solution, 50 IU/mL+50 μg/mL, respectively), gentamicin sulfate 20 μg/mL, and Fungizone (amphotericin B, 1 μg/mL). G-418 (400 μg/mL) was additionally present in GM for growth and selection of transfected clones. All of the cells were grown under an atmosphere of 5% CO_2_ in air at 37 °C in a humidified incubator until they reached 90% confluence. Then, this medium was substituted with differentiation medium DMEM with Glutamax supplemented with 4% (*v*/*v*) FBS + 2% (*v*/*v*) HS + NGF (50 ng/mL) and the same antibiotic:antimycotic mixture. Cells were allowed to differentiate for 48 h to induce neuronal phenotype and the DM was replaced by freshly prepared serum-free reference medium (RM) containing 0.1% bovine serum albumin (BSA) (*w*/*v*) + NGF (50 ng/mL) for another 24 h with or without experimental factors. When the experimental factors were dissolved in DMSO, the equivalent volume of vehicle (0.1% *v*/*v*) was added to the control cells. If necessary, the same quantity of differentiated cells with neuronal phenotype was removed from the culture plates using 0.5% (*w*/*v*) trypsin-EDTA (harvesting), centrifuged in DM at 200× *g* for 5 min, media were aspirated, and cell pellets were resuspended in RM. Media were changed every other day. Cell monolayers were harvested for Western blots or PCR. Floating dead cells were removed during media change or washed with PBS and were not included in these experiments.

### 4.2. Reagents

Atorvastatin, a cell-permeable, highly potent, and competitive inhibitor of 3-hydroxy-3-methylglutaryl coenzyme A reductase (HMGCR) was purchased from Calbiochem, Merck-Millipore (Darmstadt, Germany). Squalene synthase (SQS) inhibitor zaragozic acid (ZA), farnesyl transferase (FTase) inhibitor L-744,832, geranylgeranyl transferase I (GGTase I) inhibitor GGTI-286, simvastatin, (R)-mevalonic acid lithium salt, all-*trans*-geranylgeraniol (GGOH), farnesol (FOH), ubiquinol (UBOH), methyl-beta-cyclodextrin (MβCD), poly(ethylene-glycol 600)-cholesterol conjugate (Chol-PEG), poly(ethylene-glycol 600) (PEG), [3-(4,5-dimethylthiazol-2-yl)-2-5-diphenyltetrazolium bromide] (MTT), bovine serum albumin (BSA) were purchased from Sigma Aldrich, Saint Lois, MO, USA. C-100-Dolichol (Dolichol 20, DOH) was obtained from Larodan, Karolinska Institutet Science Park, Solna, Sweden and bisbenzimide fluorochrome from Molecular Probes, Eugene, OR, USA. Treatment of the cultures with the different experimental factors tested in the current work was performed 24 h (24 h) prior to cell harvesting, based on the respective concentration.

In order to show the protective effect of particular MEV intermediate or end-products of the MEV pathway, statins were used at a 50 μM concentration level, which significantly impaired the viability of neuronal cells (Figure 1). In turn, soluble cholesterol (1 mM, Chol-PEG) was added to restore cholesterol level previously reduced by chelation with MβCD (0.2 mM). Enzyme inhibitors were administered in concentrations recommended by the manufacturers or published elsewhere.

### 4.3. Cloning and Expression Vectors

Cloning and expression of the Homo sapiens beta-amyloid precursor protein gene 1-695 (transcript variant 3, AβPP-wt), Swedish mutation in Homo sapiens Aβ precursor protein gene AβPP-KM670/671NL (AβPP695NL), double mutation in the *AβPP* gene resulting in amino acid substitutions of Lys to Asn (codon 670) and Met to Leu (671), and PrecisionShuttle mammalian vector with C-terminal tag GFP (pCMV6-ACGFP) carrying resistance genes to antibiotics (Neor and Ampr) were ordered from OriGene Technologies, Inc., Rockville, MD, USA, and purchased from STI Bartosz Czajkowski (Poznań, Poland). Both genes (*AβPP*-wt and *AβPP*-sw) were expressed upon GFP promoter. For comparison, mock nucleofected cells (data not shown, see the reference40) underwent the complete procedure, except no vector was added prior to nucleofection.

### 4.4. Transfection of PC-12 Cells

The procedure for PC-12 cell transfection has been described elsewhere [40]. Briefly, the procedure for PC-12 cell transfection was based on the method described by the manufacturer (Amaxa Cell Line Nucleofector Kit V, Lonza Cologne AG, Cologne, Germany). Appropriate program for nucleofection of PC-12 cells was chosen form the list available in Nucleofector 4D. After 48 h in DM cells resemble neuronal phenotype (Supplementary data 1).

### 4.5. Determination of Cell Viability

Cell viability was based on the ability of cells grown on 96-multiwell plates to convert soluble MTT [3-(4,5-dimethylthiazol-2-yl)-2-5-diphenyltetrazolium bromide] into an insoluble purple formazan reaction product with minor modifications to a previously described protocol [95]. Briefly, cells were uniformly seeded in 96-multiwell flat bottomed plates and grown in GM. Confluent cultures were washed with PBS and then exposed to DM for 48 h. Afterwards, DM was replaced by RM with or without experimental factors for another 24 h. Relative viability (percentages of mean control value) was calculated. To do this, media were removed and cells were washed with PBS and further incubated with MTT (1 mg/mL) for 1 h at 37 °C in a humidified 5% CO_2_ and 95% air in incubator. Next, MTT solution was removed and water insoluble formazan was immediately dissolved in DMSO. The absorbance for MTT was measured at 490 with ELISA reader type Infinite 1000 (TECAN, Mannedorf, Switzerland). Relative percentage (versus untreated controls) of viable cells was measured by MTT conversion into purple formazan.

### 4.6. Cellular Aβ1-40 Assays

Production of Aβ1-40 was measured in PC-12 neuronal cells transfected with wild-type human *AβPP* (*AβPP*-wt, W), Swedish mutation *AβPP* (*AβPP*-sw, S) and empty vector (GFP only, G) transfected cells. Cells were seeded overnight at 3 × 10^4^ cells per well in a 96-multiwell plate. Cells were incubated in DM for 48 h, washed with PBS, and fresh RM media were added for another 24 h with or without experimental factors. Next, cellular media were harvested and assayed for the presence of Aβ1-40 with an Aβ1-40 homogenous time resolved fluorescence (HTRF) assay (CisBio) according to the manufacturer’s instructions. For all cell types (W, S and G), Aβ1-40 values were normalized for cell viability, as determined with the MTT assay.

### 4.7. Detection of Vector Sequence in PC-12-Transfected Cells by PCR

Presence of vector inserts (*AβPP*-wt or *AβPP*-sw) in PC-12-transfected cells was confirmed by PCR and described elsewhere (available online at https://www.hindawi.com/journals/bmri/2015/746092/sup/). Primers to flanking regions of TruORF were commercially delivered by the manufacturer (OriGene Technologies, Inc, Rockville, MD, USA). PCR conditions were as follows: 95 °C (2 min), 95 °C (45 s), touchdown 0.5 °C/cycle from 65 °C to 50 °C, 72 °C (1 min), 95 °C (45 s), 50 °C (45 s, 14x), 72 °C (1.5 min), 72 °C (10 min) (Table 1).

### 4.8. Western Blot Analysis

Cells, after the different treatments described above, were lysed in RIPA buffer (10 mM Na_4_P_2_O_7,_ 50 mM HEPES, 150 mM NaCl, 1% triton-X, 0.1% SDS,10 mM EDTA, 100 mM NaF, pH 7.4) containing protease inhibitor cocktail (Roche, Mannheim, Germany), insoluble material removed by centrifugation (10,000× *g*, 5 min), and protein levels quantitated with Bradford reagent (Bio-Rad, Hercules, CA, USA). Each protein extract was adjusted to a 2 μg/μL concentration added of Laemmli sample buffer (4X concentrate, Bio-Rad, Hercules, CA, USA), heated 5 min at 95 °C, and separated on precast polyacrylamide gradient gels (7.5–12%). After transfer to 0.2 μm polyvinylidene difluoride membranes (PVDF, Bio-Rad) membranes were blocked in either 5% (*w*/*v*) nonfat dried milk or 1% (*w*/*v*) BSA (Sigma Aldrich) diluted in Tris-buffered saline containing 0.1% (*v*/*v*) Tween 20 (TBS-T), and probed overnight at 4 °C with the respective primary antibodies, as follows: actin, clusterin (Santa Cruz Biotechnologies, Santa Cruz, CA, USA), AβPP, sAβPPα, beta amyloid 1–16 (6E10) (Covance, Inc., Princeton, NY, USA), AβPP (Merck Millipore, Darmstadt, Germany). Working antibody concentrations (from 1:200 to 1:2000) varied depending on the protein detected and were applied according to the manufacturer’s recommendations. Membranes were subsequently incubated with the respective HRP-linked anti-rabbit, anti-mouse IgG (Cell Signaling Technology, Danvers, MA, USA or anti-goat (Cell Signaling Technology, Danvers, MA, USA) antibodies and developed by ECL (Pierce™ ECL Western Blotting Substrate, Thermo Fisher Scientific). Densitometric quantification of band intensities was performed using analysis software (Image Studio Lite Version 5.2.5, LI-COR Biotechnology – GmbH, Bad Homburg, Germany).

## 5. Conclusions

Conclusions are summarized in the graphical abstract:

1. Statins, inhibitors of FTase, GGTase I, and SQS, as well as cholesterol chelator, reduce the viability of PC-12 neuronal cells.
2. Statin-induced neurotoxicity is overruled by GGOH, but not cholesterol administration.
3. sCLU expression is stimulated by isoprenoids and water-soluble cholesterol, however, this was not the case for GGOH administration in empty-vector cells.

## Figures and Tables

**Table 1:** Primers to flanking regions of TruORF commercially delivered by the manufacturer (OriGene Technologies, Inc, Rockville, MD, USA).

Flanking Region	Primers and their Sequences
VP1.5 (forward seq primer)	5′ GGACTTTCCAAAATGTCG 3′ *T*m = 51 ^o^C
XL39 (reverse seq primer)	5′ ATTAGGACAAGGCTGGTGGG 3′ *T*m = 60 ^o^C

## Supplementary Materials

Supplementary materials can be found at https://www.mdpi.com/1422-0067/20/6/1481/s1.

## Abbreviations

α2M	α 2 macroglobulin
AβPP	Amyloid β precursor protein
AβPP-sw	Amyloid β precursor protein with Swedish mutation
AβPP-wt	Amyloid β precursor protein wild type
Aβ	Amyloid β
AD	Alzheimer’s disease
ANOVA	Analysis of variance
ApoE	Apolipoprotein E
ATR	Atorvastatin
BBB	Blood brain barrier
Chol-PEG	Water-soluble cholesterol
CLU	Clusterin
CSF	Cerebrospinal fluid
DMPP	Dimethylalkyl diphosphate
DOH	Dolichol
FOH	Farnesol
FPP	Farnesyl pyrophosphate
FTase	Farnesyl transferase
GGOH	Geranylgeraniol
GGPP	Geranylgeranyl pyrophosphate
GGTase	Geranylgeranyl transferase
GGTI	Geranylgeranyl transferase inhibitor
HMGCR	3-hydroxy-3-methylglutaryl-CoA reductase
iCLU	Intracellular clusterin
IPP	Isopentyl pyrophosphate
LRP2	Lipoprotein low density receptor related protein 2
MβCD	Methyl-β-cyclodextrin
MEV	Mevalonate
MEVPP	Mevalonate pyrophosphate
NGF	Nerve growth factor
NOS	Nitrogen species
PC-12	Rat neuronal pheochromocytoma PC-12 cells
RAGE	Advanced glycation end products
ROS	Reactive oxygen species
sCLU	Secretory clusterin
sGTPase	Small GTP-binding protein
S.E.M.	Standard Error of Mean
SIM	Simvastatin
SQS	Squalene synthase
TLR3	Toll like receptor 3
UBOH	Ubiquinol
WB	Western blot
