# Supplementary figures and images for: Preliminary Study on Clusterin Protein (sCLU) Expression in PC-12 Cells Overexpressing Wild-Type and Mutated (Swedish) AβPP genes Affected by Non-Steroid Isoprenoids and Water-Soluble Cholesterol
Source: Int J Mol Sci. 2019 Mar 24;20(6):1481. doi: 10.3390/ijms20061481 (PMC6470582; doi:10.3390/ijms20061481)

**A**

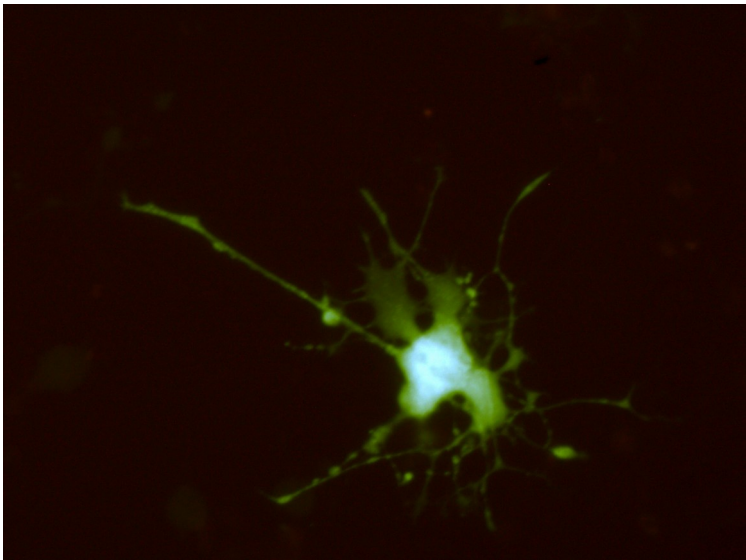

**B**

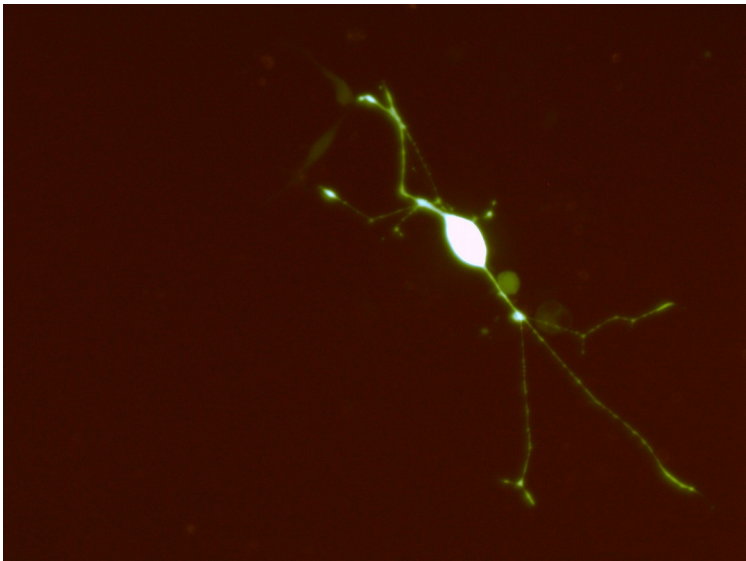

**C**

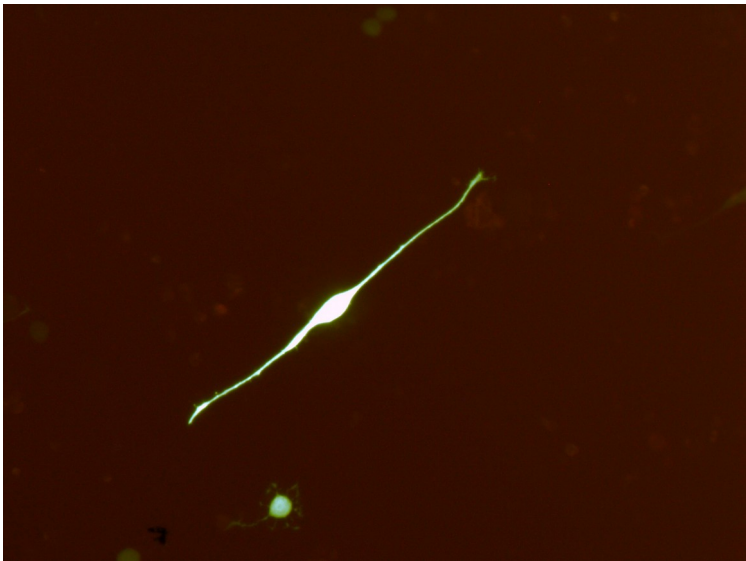

A

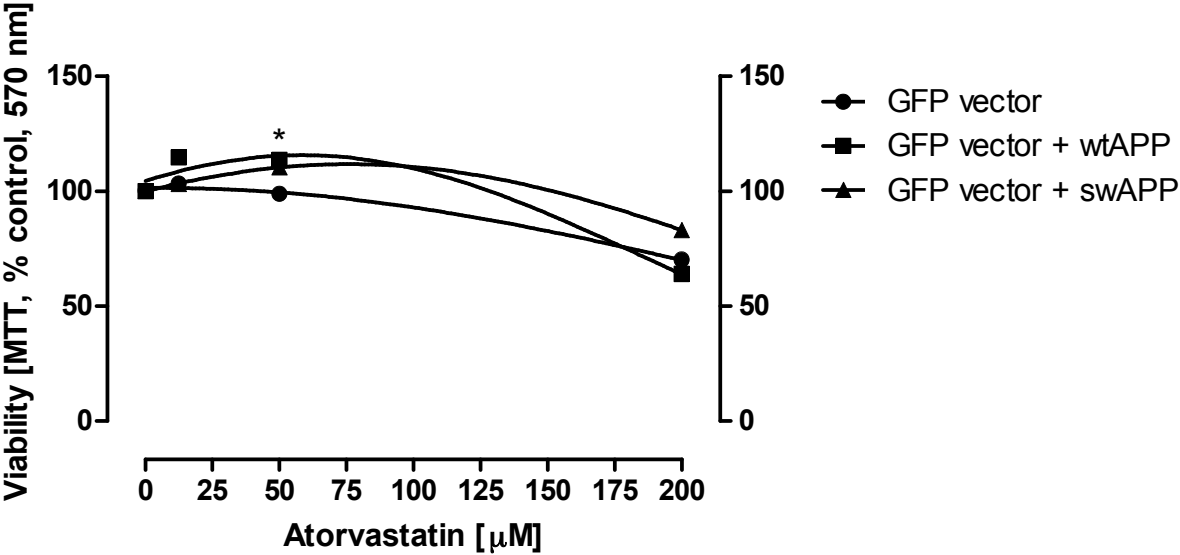

B

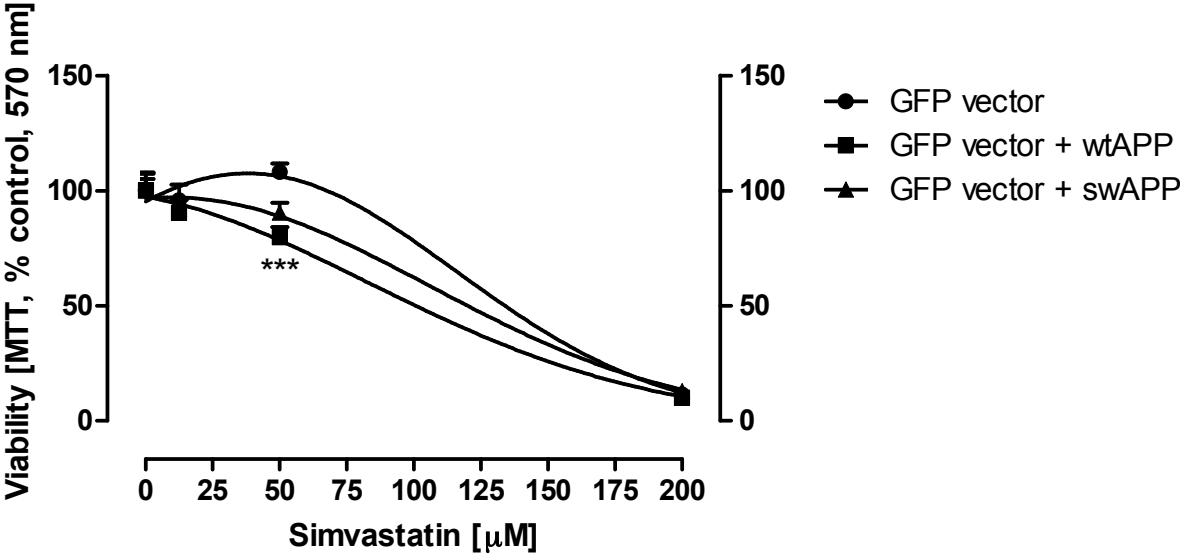

C

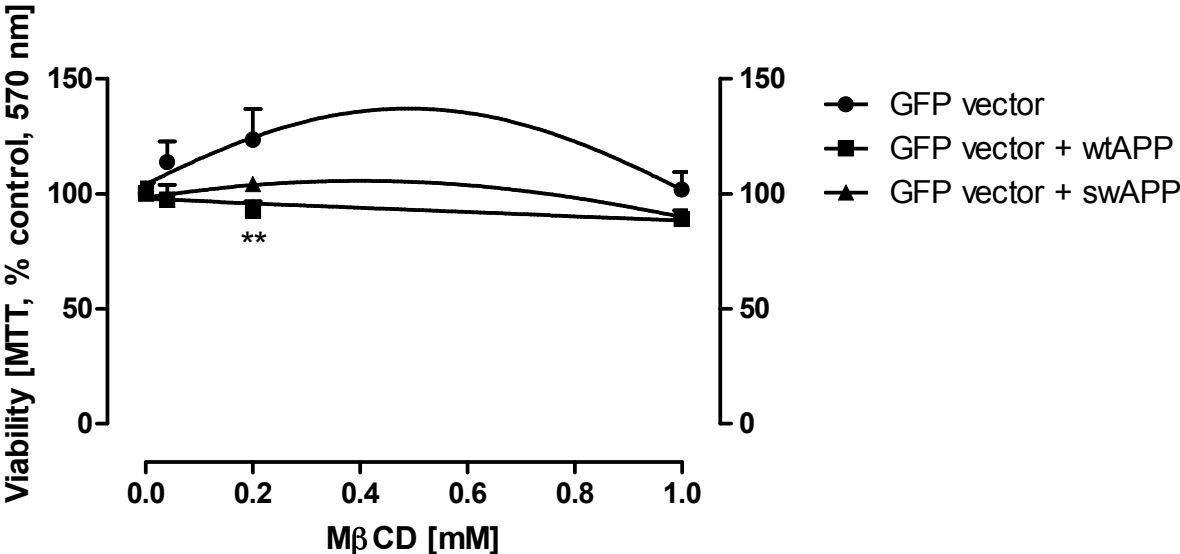

A

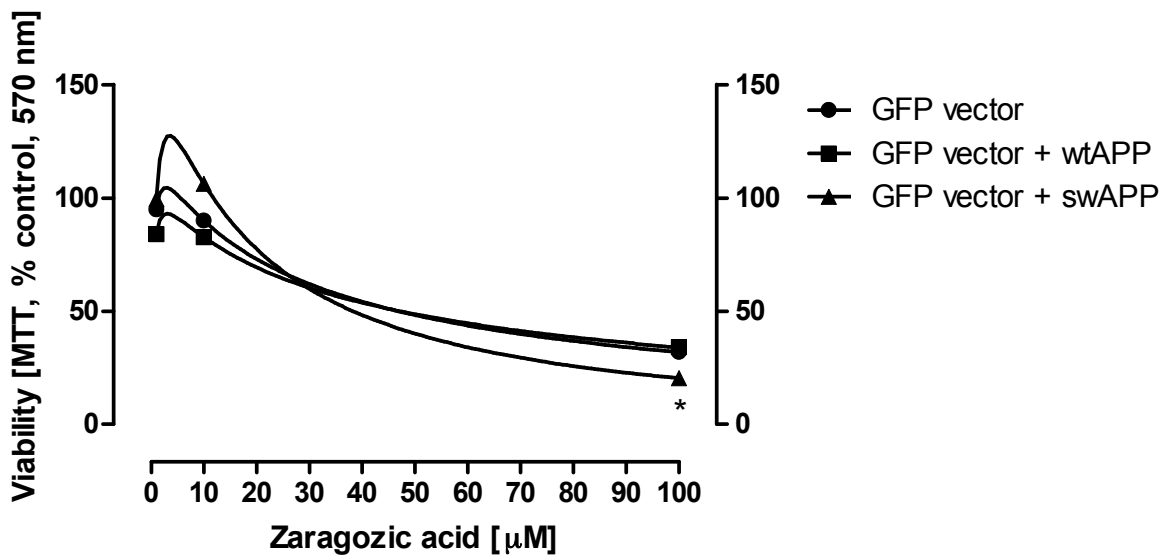

B

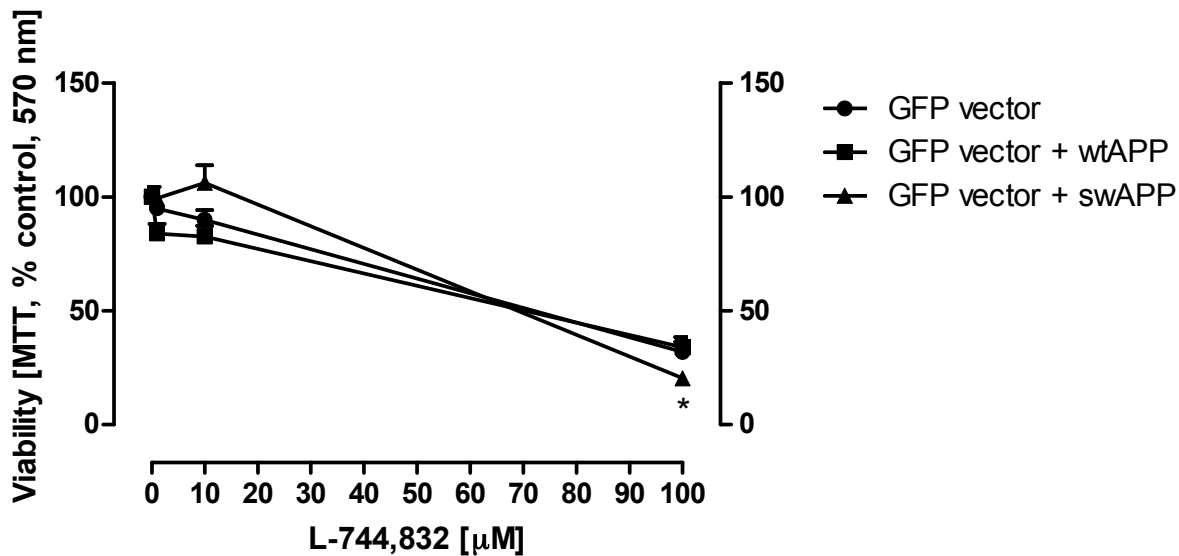

C

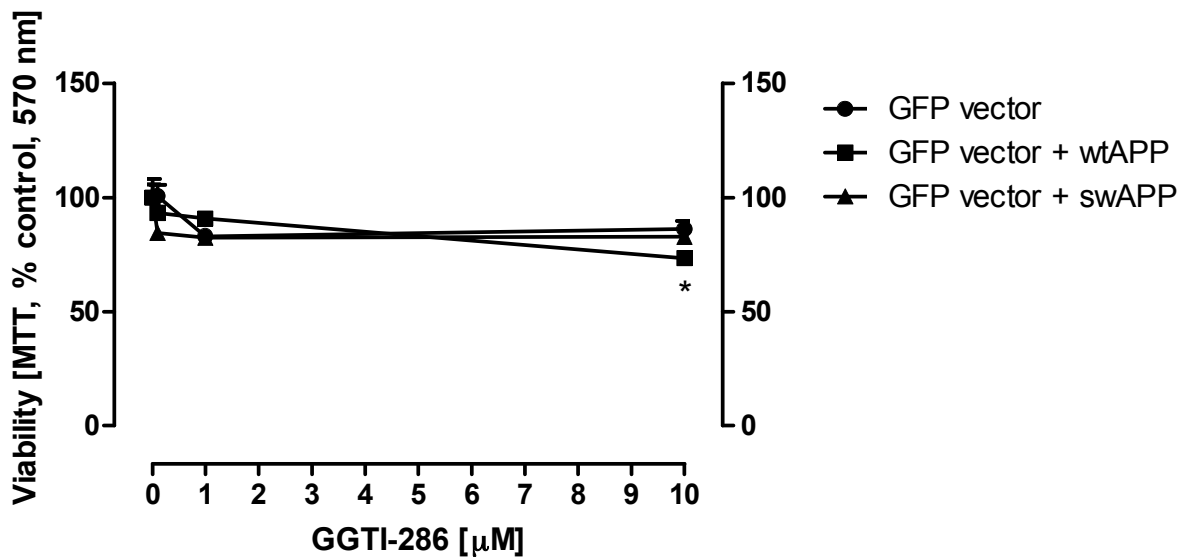

Supplement: Supplementary file 1 [file ijms-20-01481-s001.pdf]
